# Oxidative stress and inflammation cause auditory system damage via glial cell activation and dysregulated expression of gap junction proteins in an experimental model of styrene-induced oto/neurotoxicity

**DOI:** 10.1186/s12974-023-02996-3

**Published:** 2024-01-04

**Authors:** Fabiola Paciello, Anna Pisani, Rolando Rolesi, Raffaele Montuoro, Veronica Mohamed-Hizam, Giammarco Boni, Cristian Ripoli, Jacopo Galli, Renata Sisto, Anna Rita Fetoni, Claudio Grassi

**Affiliations:** 1 Department of Neuroscience, Università Cattolica del Sacro Cuore, 00168 Rome, Italy; 2 Fondazione Policlinico Universitario A. Gemelli IRCCS, 00168 Rome, Italy; 3 Department of Head and Neck Surgery, Università Cattolica del Sacro Cuore, Rome, Italy; 4 Department of Occupational and Environmental Medicine, Epidemiology and Hygiene, Italian Workers’ Compensation Authority (INAIL), Monte Porzio Catone, Rome, Italy; 5 Department of Neuroscience, Unit of Audiology, Università Degli Studi di Napoli Federico II, Naples, Italy

**Keywords:** ROS, Pro-inflammatory cytokines, Macrophages, Microglia morphology, Astrogliosis, Gap junctions, Cochlea, Auditory cortex

## Abstract

**Background:**

Redox imbalance and inflammation have been proposed as the principal mechanisms of damage in the auditory system, resulting in functional alterations and hearing loss. Microglia and astrocytes play a crucial role in mediating oxidative/inflammatory injury in the central nervous system; however, the role of glial cells in the auditory damage is still elusive.

**Objectives:**

Here we investigated glial-mediated responses to toxic injury in peripheral and central structures of the auditory pathway, i.e., the cochlea and the auditory cortex (ACx), in rats exposed to styrene, a volatile compound with well-known oto/neurotoxic properties.

**Methods:**

Male adult Wistar rats were treated with styrene (400 mg/kg daily for 3 weeks, 5/days a week). Electrophysiological, morphological, immunofluorescence and molecular analyses were performed in both the cochlea and the ACx to evaluate the mechanisms underlying styrene-induced oto/neurotoxicity in the auditory system.

**Results:**

We showed that the oto/neurotoxic insult induced by styrene increases oxidative stress in both cochlea and ACx. This was associated with macrophages and glial cell activation, increased expression of inflammatory markers (i.e., pro-inflammatory cytokines and chemokine receptors) and alterations in connexin (Cxs) and pannexin (Panx) expression, likely responsible for dysregulation of the microglia/astrocyte network. Specifically, we found downregulation of Cx26 and Cx30 in the cochlea, and high level of Cx43 and Panx1 in the ACx.

**Conclusions:**

Collectively, our results provide novel evidence on the role of immune and glial cell activation in the oxidative/inflammatory damage induced by styrene in the auditory system at both peripheral and central levels, also involving alterations of gap junction networks. Our data suggest that targeting glial cells and connexin/pannexin expression might be useful to attenuate oxidative/inflammatory damage in the auditory system.

**Supplementary Information:**

The online version contains supplementary material available at 10.1186/s12974-023-02996-3.

## Introduction

Hearing loss induced by the exposure to environmental factors, such as noise or ototoxic drugs, is primarily due to permanent alterations occurring in peripheral and central structures of the auditory pathway [1]. Indeed, hearing loss is associated with cochlear damage to hair cells, afferent neurons and synapses [2, 3] along with short- and long-term detrimental consequences in central auditory structures [4–6], with altered basal synaptic transmission and decreased spine density in the auditory cortex (ACx) [7–10]. Recent findings supported the idea that the unbalance of cellular redox status and inflammation are key mechanisms of cochlear damage. Indeed, in animal models of acquired hearing loss caused by noise exposure or ototoxic drug administration, an increase of reactive oxygen species (ROS), together with an up-regulation of inflammatory mediators have been observed in the cochlea [11–13], as well as in brain regions involved in the auditory perception [10, 14].

Dysfunction of glial cells, including astrocytes, oligodendrocytes, and microglia in the central nervous system (CNS), and Schwann cells or satellite cells in the peripheral nervous system (PNS) are strongly involved in damaging mechanisms depending on oxidative stress and inflammation [15–19].

Astrocytes play a crucial role in brain homeostasis, contributing to many functions, including blood–brain barrier maintenance, neurotransmitter cycling, metabolic and synaptic support [20–22]. Similarly, microglia, the resident innate immune cells in the CNS [23, 24], regulates several brain functions, such as synaptic plasticity and synaptogenesis, and shapes neuronal circuits during development [25, 26], including auditory brainstem pathways [27], and in post-natal life [28]. Among microglia general markers, ionized calcium-binding adapter molecule 1 (IBA-1), cluster of differentiation receptors, such as CD68, and fractalkine receptor (CX3CR1) are the most widely used, although expressed also in macrophages [20, 21, 29–31]. Whereas, largely specific microglia markers include transmembrane protein 119 (TMEM119) and purinergic receptor P2YR12 [29, 30].

Macrophages are phagocytic cells, considered the major overseers of the immune system, regulating tissue homeostasis and inflammatory responses [32–34]. They are generally classified in tissue-resident macrophages, responsible for tissue repair, homeostasis, and inhibition of inflammation [35, 36], and monocytes, recruited from the bone-marrow following an insult, promoting the inflammatory response [36–38]. Thus, in the auditory system, macrophages have been found distributed in both the peripheral organ, the cochlea (macrophages and perivascular macrophage-like melanocyte) and in the CNS (microglia and perivascular macrophages in the cerebral cortex) [38].

Different from microglia, arising from erythro-myeloid precursors in the yolk sac and, during early stages of embryonic development, migrating to the CNS [39, 40], macrophages are derived from monocytes, which originate from bone-marrow hematopoietic stem cells [41]. On a functional point of view, macrophages and microglia are both plastic, adapting their phenotype to the local tissue micro-environment by changing their morphology, abundance, and distribution in response to insults [32, 33, 38, 42, 43].

Following an injury, glial cells start modulating the production of reactive oxygen and nitrogen species. Indeed, microglia is involved in increased ROS generation in the CNS [44–46] and both microglia and astrocytes can modulate inflammatory responses through cytokines, chemokines, and oxidative stress markers [47, 48]. In this view, several studies underlined the role of gap junctions (GJCs), hemichannels (HCs) and pannexons in affecting glial cell function and dysfunction [49–53]. Microglial and astrocyte activation is a complex and dynamic process, based on molecular signaling pathways occurring through opening GJCs, HCs and pannexin (Panx) channels [52, 54, 55]. On the other hand, activated microglia affect connexin expression and GJC and HC functional activity in astrocytes [56–61].

Studies performed in different animal models reported persistent macrophages activation in the cochlea and in the auditory nuclei of brainstem, specifically the cochlear nucleus, after auditory deprivation caused by cochlear deafferentation [62–65]. However, although the contribution of microglia and astrocytes to neuroinflammatory damage has been documented in several CNS disorders, less is known about their role in the auditory system. Thus, we studied immune and glial cell responses in the auditory peripheral organ (i.e., the cochlea) and in the ACx using an experimental model of oto/neurotoxic damage caused by the administration of styrene, a volatile organic solvent with well-documented ototoxic effects at occupational level [66–69].

Here, we report that macrophages activation in the cochlea and glial cell response in the ACx play a critical role in the oxidative/inflammatory damage of the auditory system, also involving altered expression of connexins and pannexins in cochlear and brain regions.

## Materials and methods

### Animals

**Fig. 1:**
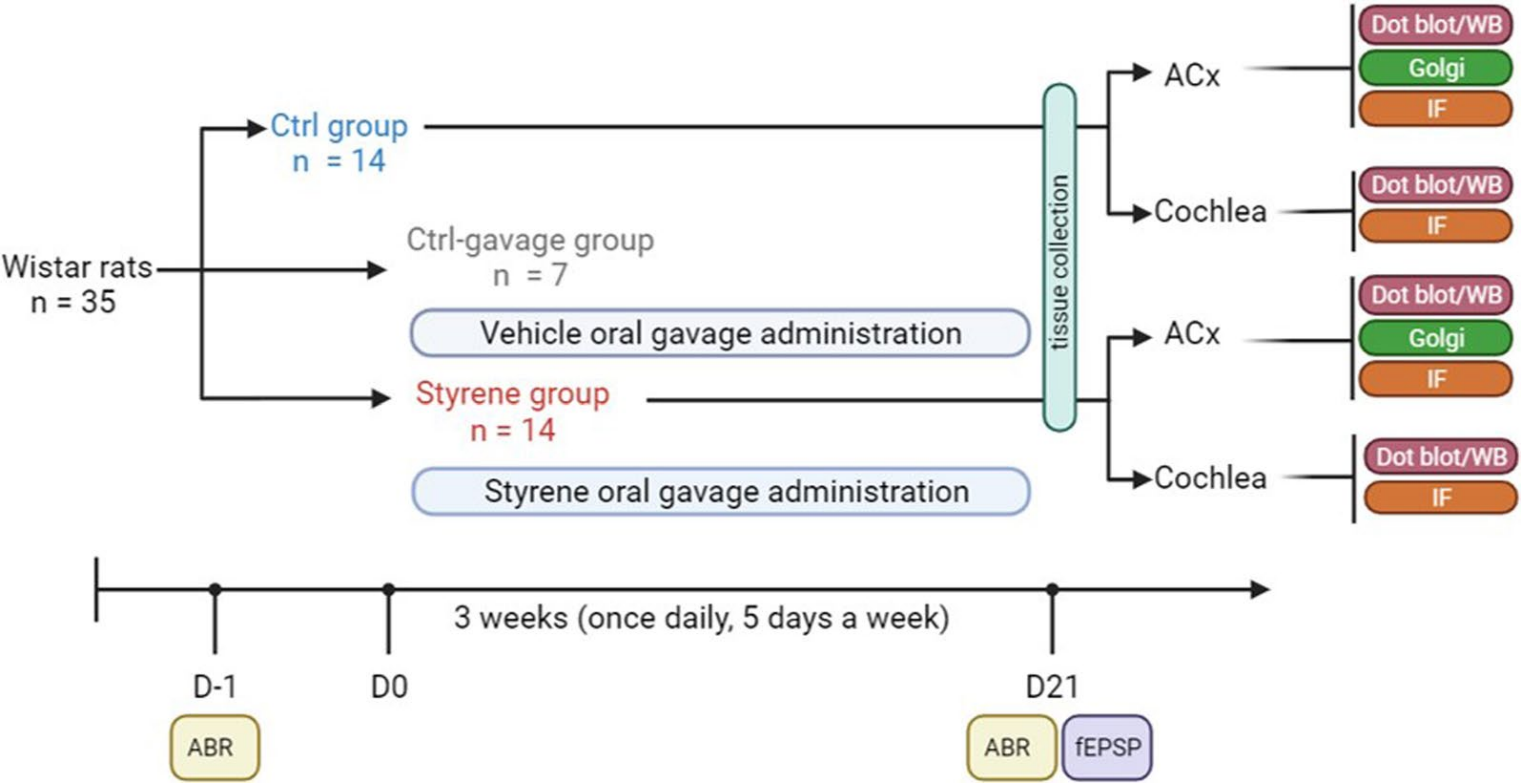
**Workflow of the experimental design.** Male adult Wistar rats were randomly assigned at the beginning of the study to three experimental groups: “Ctrl”, “Styrene” and “Ctrl-gavage” groups. Baseline hearing thresholds were evaluated in all animals the day (D) before starting styrene treatment (D-1) by recording auditory brainstem responses (ABR). Animals of Styrene group underwent oral gavage treatment with styrene (400 mg/kg) for 3 weeks (from D0 to D21), once daily, 5 days a week. At the same time, Ctrl-gavage group underwent vehicle delivery (olive oil). At the end of the treatment (D21), after ABR recordings, animals were scarified and tissues, both cochlea and auditory cortex (ACx), were collected to perform experimental analyses. fEPSP: field excitatory postsynaptic potentials; WB: western blot; IF: immunofluorescence; Golgi: Golgi–Cox staining

In our experimental plan, we used *n* = 35 male adult Wistar rats (2–3 months of age) with normal Preyer’s reflex, randomly assigned to three experimental groups: (1) control animals (“Ctrl” group; *n* = 14); (2) animals treated with styrene by oral gavage (“Styrene” group; *n* = 14) and (3) control animals that underwent oral gavage of olive oil (“Ctrl-gavage” group; *n* = 7). Rats were housed two per cage with free access to food (Mucedola 4RF21, Italy) and water. Housing environment was temperature (22–23 °C) and humidity (60% ± 5%) controlled, with a 12-h light/dark cycle. All efforts were made to minimize animal suffering and number, in agreement with the European Community Council Directive of 24 November 1986(86/609/EEC). All procedures were performed in accordance with the Laboratory of Animal Care and Use Committee of the Catholic University, School of Medicine of Rome and were approved by the Italian Department of Health (*Ministero della Salute*, Prot. 1F295.116 and Prot. 1F295.117). A schematic representation of the experimental plan is reported in Fig. 1. A list of abbreviations in the whole text is reported in the Additional file 1: Table S1. Experiments were performed according to the Arrive guidelines (Additional file 2).

### Oto/neurotoxic insult: styrene administration

To damage the auditory system, we used styrene administration, a well-known oto/neurotoxic volatile compound. Indeed, styrene exposure has been previously shown to cause cochlear damage and hearing loss [66, 67]. Thus, styrene (styrene > 99%, Sigma Corporation, product id: S4972) was dissolved in olive oil, as reported in previous published protocols [66, 67] and administered by oral gavage for 21 days, following a dosage and time schedule of treatment previously used (400 mg/kg daily for 3 weeks, 5/days a week) [66, 67, 69]. At the end of styrene treatment, animals were sacrificed, brain and cochlear samples were collected to perform all experimental evaluations.

### Auditory brainstem responses (ABR)

To evaluate cochlear damage and hearing loss we measured ABRs, as described previously [8]. Briefly, using active, reference and ground needle electrodes (inserted subcutaneously in tested pinna, vertex, and contralateral pinna, respectively), we recorded auditory bioelectrical potentials in animals mildly anesthetized (ketamine, 35 mg/kg and medetomidine-domitor, 0.25 mg/kg) subjected to acoustic click stimuli at decreasing intensity levels (from 100 to 10 dB). Data were collected and analyzed using a TDT System 3 (Tucker Davis Technologies, Alachua, FL, United States).

The lower stimulus intensity able to evoke a repeatable ABR waveform was considered as the auditory threshold. We also measured the wave II amplitude–intensity (A–I) curve from ABR recordings as previously reported [7, 70].

### Ex vivo electrophysiology

We performed field recordings on coronal slices (400 μm-thick) containing the ACx as previously described [8–10]. FEPSPs were evoked in pyramidal neurons of layer II/III of the ACx by stimulating local connections using a concentric bipolar tungsten electrode (FHC Inc., Bowdoin, ME, USA) connected to a stimulator. I/O curves were obtained by afferent fiber stimulation at intensities ranging from 0 to 300 µA (in increments of 50 µA; stimulus rate of 1 pulse every 20 s).

### Tissue section preparation and immunofluorescence staining

Immunofluorescence experiments were performed in both cochlear and ACx samples.

In cochlear specimens, we performed immunofluorescence analyses in both cryosections (obtained as described in [66]) and in surface preparations of the basilar membrane with the organ of Corti, obtained through cochlear microdissection as described previously [7, 8].

To perform analyses in the ACx we used 40-μm-thick coronal brain cryosections, collected from 2.18 mm to 3.4 mm posterior to bregma [71], containing the ACx [8, 10]. Cochlear surface preparations/sections or brain sections containing ACx were incubated with a blocking solution (containing 1% BSA, 0.5% Triton X-100 and 10% normal goat serum in PBS 0.1 M) and then they were incubated overnight at 4 °C with a solution containing primary antibodies against: IBA-1, GFAP, COX-2, CtBP2, NF200, CD68. All specimens were incubated at room temperature (RT) for 2 h with donkey anti-rabbit and/or anti-mouse secondary antibody solution (Alexa Fluor 488 or 546) and DAPI stained to visualize cell nuclei. Detailed information about the antibodies is reported in Table 1.

**Table 1:** List of reagents and antibodies used

Reagent type	Designation	Source	Identifiers	Additional information	Use
Antibody	Anti-IBA-1(rabbit monoclonal)	Cell signaling	Cat. No. #17198	IF (1:100)	Marker of microglia/macrophages
Antibody	Anti-rabbit IgG AlexaFluor 546 (rabbit polyclonal)	Thermo fisher scientific	Cat. No. #A-10040	IF (1:400)	Secondary antibody for immunofluorescence analyses
Antibody	Anti-rabbit IgG AlexaFluor 488 (rabbit polyclonal)	Thermo fisher scientific	Cat.No. #A-21206	IF (1:400)	Secondary antibody for immunofluorescence analyses
Antibody	Anti-mouse IgG AlexaFluor 546 (mouse polyclonal)	Thermo fisher scientific	Cat.No. #A10036	IF (1:400)	Secondary antibody for immunofluorescence analyses
Nuclear Counterstains	DAPI	Thermo fisher scientific	Cat.No. D1306	IF (0.5 mg/mL)	Marker of cell nuclei
Antibody	Anti-GFAP (mouse monoclonal)	Cell signaling	Cat. No. #3670	IF (1:300) WB (1:1000)	Marker of glial fibrillary acidic protein (glial cells and astrocytes)
Antibody	Anti-COX-2 (rabbit monoclonal)	Cell signaling	Cat. No. #12282	IF (1:500) WB (1:1000)	Marker of cyclooxygenase2 protein (inflammatory marker)
Antibody	Anti-CtBP2 (mouse)	BD biosciences	Cat. No. 612044	IF (1:1000)	Marker of C-terminal-binding protein 2 (pre-synaptic contact)
Antibody	Anti-NF200 (rabbit polyclonal)	Sigma-Aldrich	Cat. No. N4142	IF (1:80)	Marker of neurofilaments (afferent fibers)
Antibody	Anti-CD68 (mouse monoclonal)	Abcam	Cat. No. ab955	WB (1:1000) IF (1:100)	Phagocytic markers involved in microglia and macrophages activation
Antibody	Anti-cleaved-caspase 3 (rabbit polyclonal)	Millipore	Cat. No. #AB3623	WB (1:100)	Marker of cleaved-caspase-3 protein (active form in apoptotic pathway)
Antibody	Anti-Caspase-3 (rabbit polyclonal)	Santa cruz biotechnology	Cat. No. sc-7272	WB (1:1000)	Marker of total caspase-3 protein
Antibody	Anti-NF-κB (rabbit monoclonal)	Cell signaling	Cat. No. #8242	WB (1:1000)	Transcription factors of the nuclear factor κB (NF-κB) (inflammatory marker)
Antibody	Anti-TNF-α (mouse monoclonal)	Santa cruz biotechnology	Cat. No. sc-52746	WB (1:1000)	Tumor Necrosis Factor α (inflammatory marker)
Antibody	Anti-IL-1β (rabbit polyclonal)	Santa cruz biotechnology	Cat. No. sc-7884	WB (1:1000)	Interleukin-1 β (IL-1β) (inflammatory marker)
Antibody	Anti-CXCR1 (rabbit polyclonal)	Thermofisher	Cat. No. #PA5-95749	WB (1:1000)	Marker of chemokine receptors protein
Antibody	Anti-iNOS (rabbit polyclonal)	Thermofisher	Cat. No. # PA1-036	WB (1:1000)	Marker of Inducible Nitric Oxide Synthase (iNOS)
Antibody	Anti-Cx43 (rabbit polyclonal)	Abcam	Cat. No. ab62252	WB (1:8000)	Marker of Connexin-43
Antibody	Anti-Cx26 (mouse monoclonal)	Thermofisher	Cat. No. # 13-8100	WB (1 µg/ml)	Marker of Connexin-26
Antibody	Anti-Cx30 (rabbit polyclonal)	Thermofisher	Cat. No. #71-2200	WB (1 µg/ml)	Marker of Connexin 30
Antibody	Anti-Panx1 (rabbit polyclonal)	Thermofisher	Cat. No. #487900	WB (1 mg/ml)	Marker of Pannexin 1
Antibody	Anti-Nitro tyrosine (rabbit)	Cell signaling	Cat. No. #06-284	WB (1:1000)	Marker of protein tyrosine nitration
Antibody	Anti-4HNE (rabbit polyclonal)	Alpha diagnostic international	Cat. No. #HNE11-S	WB (1:1000)	Detect the 4-Hydroxy-2-nonenal (HNE) a marker of lipid peroxidation
Antibody	Anti-GAPDH (mouse monoclonal)	Abcam	Cat. No. ab8245	WB (1:10,000)	Loading control
Antibody	Anti-β-tubulin (mouse monoclonal)	Cell signaling	Cat. No. #2146	WB (1:5000)	Loading control
Antibody	Anti-phospho-AMPA Receptor 1 (GluA1, Ser845) (rabbit polyclonal)	Cell signaling	Cat. No. #8084	WB (1:1000)	Receptor function, trafficking, and conductance
Antibody	Anti-AMPA Receptor 1 (GluA1) (rabbit polyclonal)	Cell signaling	Cat. No. #13185	WB (1:1000)	Total GluA1 levels

To evaluate ROS production in the ACx we used dihydroethidium (DHE) staining as described in [9]. Fluorescent images were acquired using a confocal laser scanning microscope (Nikon Ti-E, Confocal Head A1 MP, Tokyo, Japan) with a 20 × objective lens.

To verify the specificity of fluorescence labeling, control experiments were obtained by omitting the primary antibodies in samples randomly selected (data not shown).

### Morphological analyses in the ACx: glial cell morphology and neuronal spine density

To evaluate neuronal dendritic spine density in ACx pyramidal neurons of layer II/III, brains from Styrene and Ctrl animals (*n* = 3/group) were stained with the Golgi–Cox solution, according to previously published protocol [7–9]. Images were collected and analyzed by an Olympus BX63 microscope, equipped with a 100 × objective.

Macrophages, microglial and astrocyte cells were identified by immunofluorescence staining using IBA-1 and GFAP-specific antibodies to analyze cell morphology [72–74]. Thus, IBA-1 and GFAP positive-cells in the ACx were visualized with a Zeiss microscope with a motorized stage, connected to Neurolucida 7.5 software (MicroBright-Field). To analyze cell morphology, Sholl analysis was applied [75]. Only cells clearly detectable and displaying intact processes were taken into consideration. We analyzed several structural parameters, including: (1) process length (in µm); (2) the number of branch points (bifurcating nodes); (3) the total number of intersections between process and a shell and (4) average of total surface area in µm^2^ [76–78].

### Western immunoblot and dot blot analyses

To obtain semi-quantitative data on protein levels, we performed western blots or dot blots on both cochlear and ACx lysates, obtained from at least 3/animals/group, as previously described [10, 79]. Briefly, total proteins were extracted using ice-cold RIPA buffer according to our previous published protocols [10, 80] and protein lysates (30 μg) were loaded onto Tris–glycine polyacrylamide gels for electrophoretic separation. Proteins were then transferred onto nitrocellulose membranes and, after 1 h with blocking buffer (5% skim milk in TBST), they were incubated overnight at 4 °C with primary antibodies. Membranes were then incubated overnight at 4 °C with a solution containing antibodies against: caspase, cleaved-caspase 3, NF-κB, TNF-α, IL-1β, COX-2, CXCR1, GFAP, CD68, iNOS, Cx43, Cx26, Cx30, Panx1.

For dot blot, 5 μl of cochlear or ACx lysates (5 μg/μl) were spotted into a TBST pre-wetted nitrocellulose membrane as described previously [9]. Antibodies against 3-nitrotyrosine (3-NT), and 4-hydroxynonenal (4-HNE) were used to evaluate protein tyrosine nitration and lipid peroxidation, respectively.

After incubation with primary antibody solution, western blot or dot blot membranes were washed in TBST and then incubated with HRP-conjugated mouse or rabbit secondary antibodies for 1 h at RT. Primary antibodies anti-GAPDH, or anti-β-tubulin mouse monoclonal antibody were used to make sure of equal protein loading. Detailed information about all antibodies used is reported in Table 1.

The quantification of protein levels was performed using UVItec Cambridge Alliance system. Experiments were performed in triplicate.

### Statistical analysis

Sample size was determined after a power analysis performed to obtain a statistical power of 80% at an α level of 0.05. Statistical analyses were performed in a blind manner. The statistical tests used (two-way ANOVA or Student’s *t* test) are specified in the main text and in figure legends. Tukey’s *Post-hoc* was used for multiple comparisons (SigmaPlot 14.0 or Statistica, Statsoft). The level of significance was set at 0.05. Results are presented as mean ± SEM.

## Results

### Styrene exposure causes functional and morphological damage in the auditory system

**Fig. 2:**
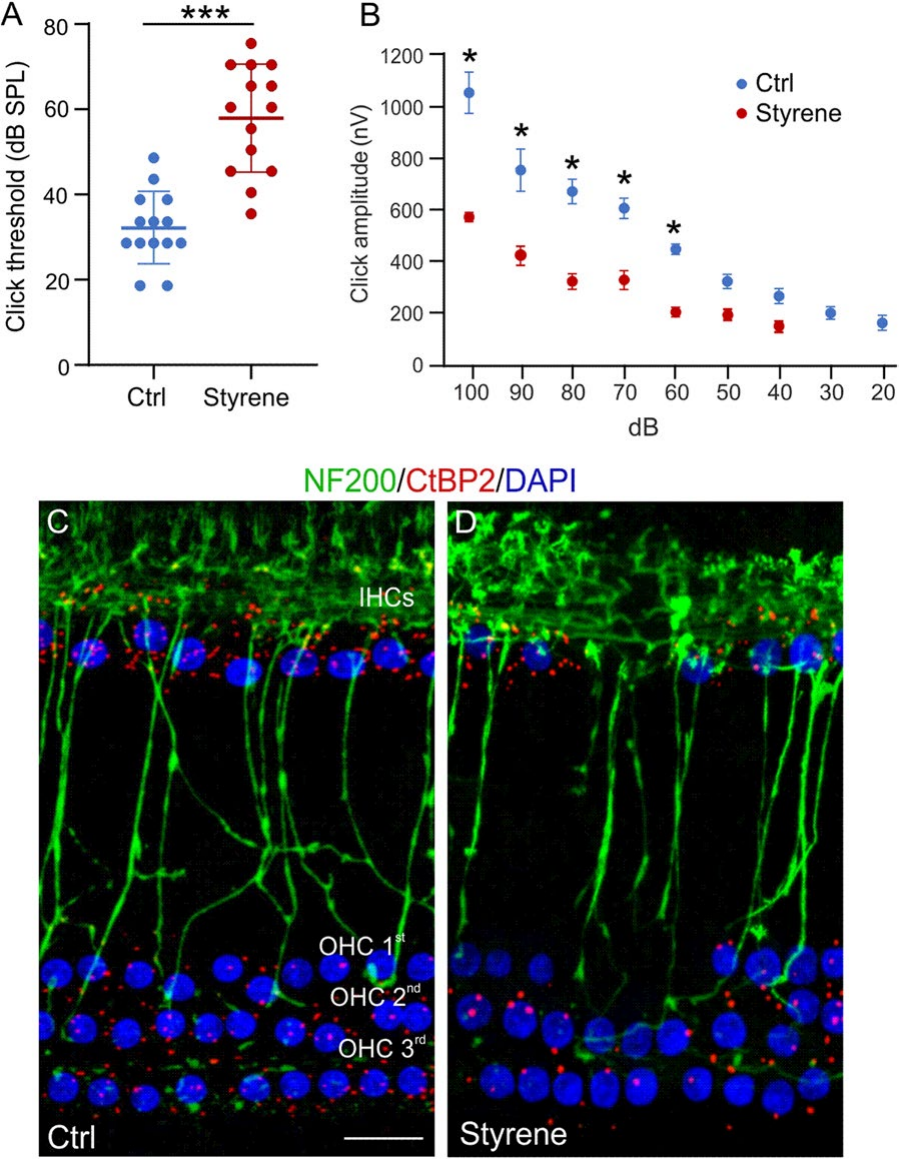
**Functional and synaptic damage caused by styrene exposure in the cochlea. A** Graph show mean ABR threshold values (means ± SEM) for click stimuli indicating a hearing loss (of about 25 dB) after styrene treatment (*n* = 14 rats for each group; Student’s *t* test, *p* < 0.001). **B** Amplitude–intensity curves obtained by measuring amplitude of wave II of ABR click responses with decreased intensity (means ± SEM). Styrene treatment induces a significant decrease of wave II amplitude, confirming neuronal damage. **C**, **D** Images of surface preparations of the organ of Corti from control (**C**) and styrene-treated animals (**D**). The one row of inner hair cells (IHCs) and three rows of outer hair cells (OHCs) are stained with DAPI to visualize cell nuclei. CtBP2 puncta (red staining) indicate pre-synaptic contacts between the hair cells and afferent nerve fibers, stained with NF200 (in green). Scale bar: 20 μm. Significant differences between groups are indicate by asterisks (***p < 0.001)

To determine the role of microglia and astrocyte activation in the auditory system damage we used an experimental model of oto/neurotoxic injury caused by styrene administration. Figure 1 summarizes all experimental procedures and a timeline of the experimental plan. We previously reported that styrene exposure impinges on cochlear function, leading to hearing loss and diminished neuronal transmission times in primary afferent fibres [66, 67]. Here, we first confirmed our previous findings, showing that styrene administration induced an increase of auditory threshold of about 25 dB in click responses at the end of treatment (day 21, Fig. 2A; *n* = 14 rats for each group; Student’s *t* test, *p* < 0.001). No significant differences were found between Ctrl and Ctrl-gavage in ABR values (data not shown), indicating no alteration of auditory function. The A–I curve derived from ABR wave II amplitude, for click responses at different intensity levels (dB), showed a significant decrease of amplitude responses in Styrene animals, compared to Ctrl group (Fig. 2B; *n* = 14 rats for each group; Student’s *t* test, *p* < 0.001). This functional injury was also associated with cochlear synaptic damage. Indeed, our immunofluorescence analysis on surface preparations of the organ of Corti showed fewer synaptic ribbons (Fig. 2C, D) in both outer (OHCs) and inner (IHCs) hair cells, associated with reduced number of primary afferent fibres in styrene-treated animals compared to controls (compare Fig. 2C with Fig. 2D).

**Fig. 3:**
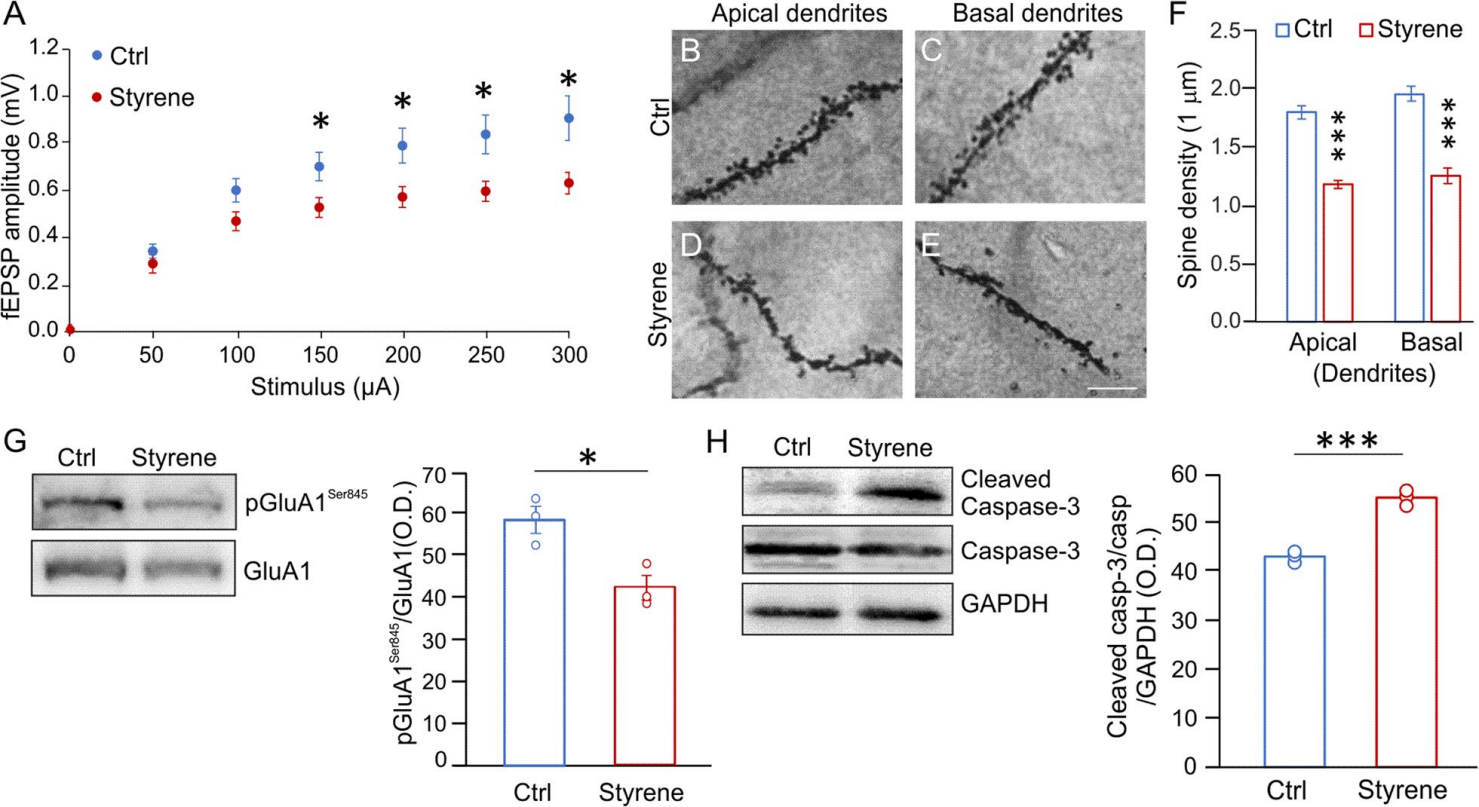
**Morphological and functional damage induced by the oto/neurotoxic insult in the auditory cortex (ACx). A** Graph shows results of field excitatory post-synaptic potential (fEPSP) amplitude measured following stimulation of afferent fibers in ACx layer II/III at increasing intensities. Statistical analysis by two-way ANOVA followed by Tukey’s post-hoc revealed significant differences between groups (*p* < 0.001; *n* = 17 slices from 4 Styrene and *n* = 16 slices from 4 Ctrl rats). **B**–**E** Representative images of Golgi-stained segments from apical (**B**, **D**) and basal dendrites (**C**, **E**) of pyramidal neurons of layers II/III in Ctrl and Styrene groups. Scale bar: 10 μm. **F** Bar graphs showing values of spine density in apical and basal dendrites of neurons of layer II/III of the ACx in the experimental groups (*n* = at least 30 segments from 30 different neurons were analyzed from three animals/groups; two-way ANOVA, apical dendrites *p* < 0.0001, basal dendrites *p* < 0.0001). **G**, **H** Images of western immunoblot indicating lower pGluA1^Ser845^ (**G**) and higher cleaved caspase-3 (**H**) levels in the ACx of styrene-treated rats compared to controls. Histograms show densitometric analyses in all samples normalized to total protein amount (GluA1 and GAPDH/Caspase-3) (*n* = 3 animals for each group; Student’s *t* test, pGluA1 *p* = 0.022; Caspase-3 *p* = 0.0004). Data are expressed as mean ± SEM. Asterisks indicate statistical significance (*p < 0.05; ***p < 0.001)

Moving to the ACx, we studied synaptic responses after focal stimulation of ACx layer II/III neurons in slices from Styrene and Ctrl animals. Comparing the I/O curves, we observed that fEPSPs were significantly lower in rats exposed to styrene compared to controls (Fig. 3A; *n* = 17 slices from 4 Styrene and *n* = 16 slices from 4 Ctrl rats; two-way ANOVA, Tukey’s post-hoc test, F_(1,217)_ = 28,473, *p* < 0.001), indicating that basal synaptic transmission in ACx was altered by the toxic insult. At morphological level, we performed spine density analysis in ACx layer II/III neurons. We found a significant decrease of spine density in both neuronal apical and basal dendrites of styrene-treated animals with respect to controls (Fig. 3B–F, Student’s *t* test, *p* < 0.0001; Ctrl *n* = 40 segments from *n* = 3 rats/group and Styrene *n* = 30 segments analysed from *n* = 3 rats/group), indicating that the auditory toxic insult affected glutamatergic synapses in the ACx. Thus, at molecular level, we focused on the AMPA receptor phosphorylation (AMPAR) at Ser845 (pGluA1^Ser845^), which is known to be crucial in receptor function, trafficking, and conductance, also in experimental models of experience-dependent synaptic plasticity [81–84]. Western blot analyses on ACx lysates revealed a significant low level of pGluA1^Ser845^ in styrene-treated animals (Fig. 3G, *n* = 3 animals/group; Student’s *t* test, *p* = 0.022), confirming altered glutamatergic transmission. Finally, we found a significant high level of cleaved caspase-3 in ACx lysates of styrene-treated animals compared to controls, indicating the activation of apoptotic pathway (Fig. 3H, *n* = 3 animals/group; Student’s *t* test, *p* = 0.0004).

### Oxidative stress and neuroinflammation in the cochlea and auditory cortex of styrene-treated animals

**Fig. 4:**
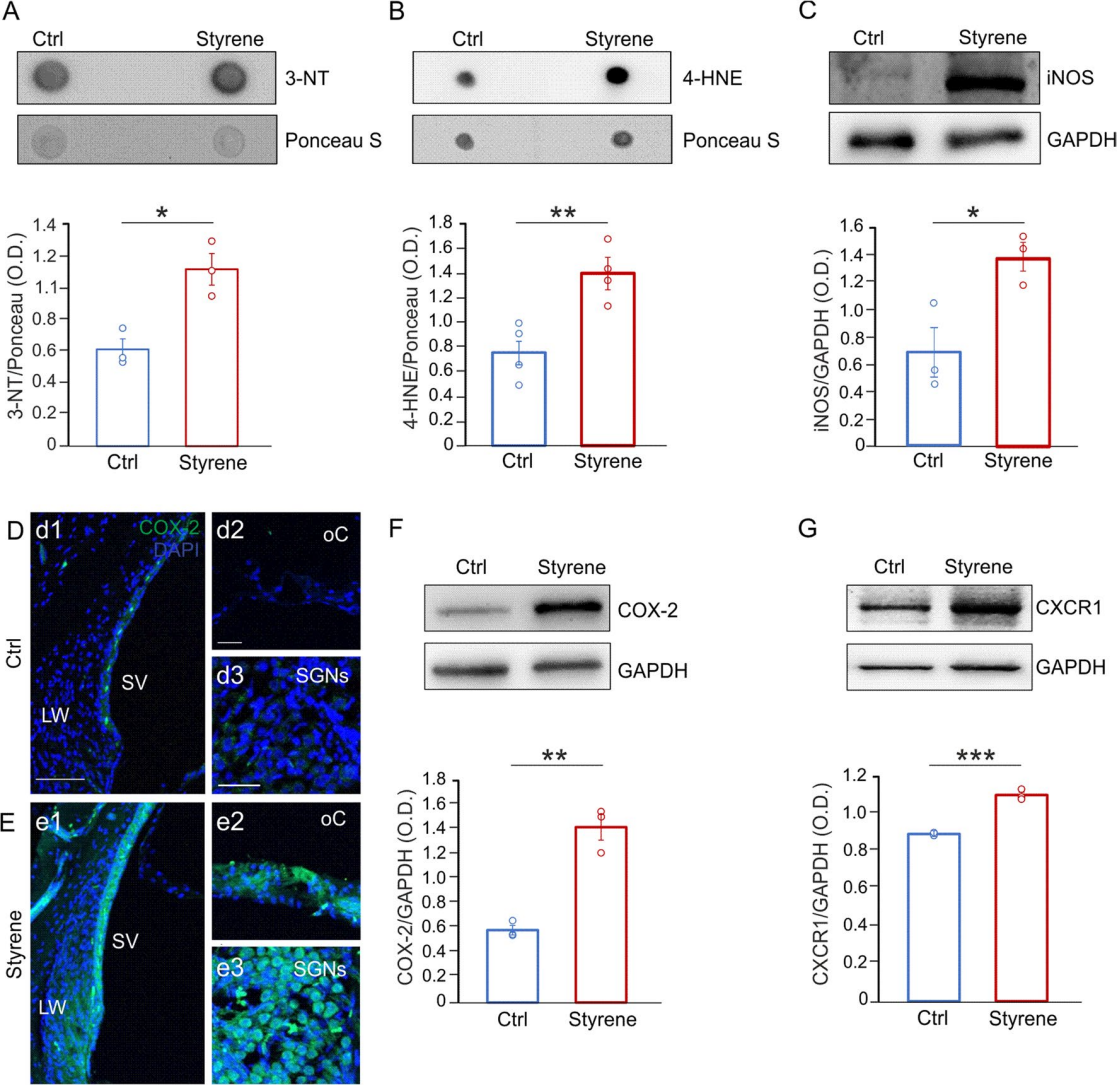
**Increased oxidative stress and neuroinflammation in the cochlea after styrene exposure. A**, **B** Representative dot blots showing high protein tyrosine nitration (3-NT, *n* = 3 rats for each group; Student’s *t* test, *p* = 0.013) and lipid peroxidation (4-HNE, *n* = 4 animals for each group; Student’s *t* test, *p* = 0.006) in the cochlea of styrene-treated animals compared to control samples. Ponceau S staining confirmed equal protein loading. **C** Western blot images showing elevated levels of iNOS, indicating oxidative damage in the cochlea of styrene-treated animals compared to controls (*n* = 3 animals for each group; Student’s *t* test, *p* = 0.029). **D**, **E** Representative images of cochlear longitudinal sections showing high magnifications of the lateral wall (LW) with stria vascularis (SV; **d1**, **e1**), the organ of Corti (oC; **d2**, **e2**) and spiral ganglion neurons (SGNs; **d3**, **e3**) stained with COX-2, as a marker of oxidative-inflammatory damage (green fluorescence) and DAPI (blue fluorescence) to label cell nuclei. A marked increase of fluorescence signal was observed in Styrene (**E**) compared to Control (Ctrl) group (**D**). Data are representative of three independent experiments from three animals/group. Scale bar: 100 μm in **d1**; 30 μm in **d2** and 50 μm in **d3**. **F**, **G** Western blot bands showing high levels of COX-2 (**F**) and CXCR1 (**G**) in Styrene group compared to Ctrl group. Histograms (means ± SEM) show data from densitometric analyses on all samples (COX-2 *n* = 3 animals for each group; Student’s *t* test, *p* = 0.001; CXCR1 *n* = 3 animals for each group; Student’s *t* test, *p* = 0.0002) normalized to GAPDH. Asterisks indicate significant differences between groups (*p < 0.05; **p < 0.01; ***p < 0.001)

Previous studies, including ours, demonstrated redox imbalance associated with inflammation in the cochlea following styrene exposure [66, 67, 85]. Here, we extended our analyses to the whole auditory system by also studying central auditory brain structures, i.e., the ACx. First, we performed dot blot analyses showing high level of 4-HNE and 3-NT in Styrene cochlear samples (Fig. 4A, B, 3-NT *n* = 3 animals/group; Student’s *t* test, *p* = 0.013; 4-HNE *n* = 4 animals/group; Student’s *t* test, *p* = 0.006), thus indicating high level of protein tyrosine nitration and lipid peroxidation in animals treated with the toxic compound. Moreover, we studied nitric oxide (NO) production by analysing the level of inducible NOS (iNOS) that was significantly higher in cochlear samples of animals treated with styrene (Fig. 4C, *n* = 3 animals/group; Student’s *t* test, *p* = 0.029), with respect to controls. These findings confirmed that styrene cochlear injury involves oxidative damage, altering cochlear redox status. We also evaluated the cochlear level of cyclooxygenases-2 (COX-2), usually observed in response to inflammatory stimuli [86], by immunofluorescence and western blot analyses. Our results showed a marked COX-2 fluorescence in styrene-treated animals (Fig. 4E) compared to control specimens (Fig. 4D), specifically in the stria vascularis (Fig. 4d1, e1), the organ of Corti (Fig. 4d2, e2) and SGNs (Fig. 4d3, e3). The COX-2 high level in the cochlea of styrene-treated animals was also confirmed by western blot analyses (Fig. 4F, *n* = 3 animals/group; Student’s *t* test, *p* = 0.001). Finally, we focused on the chemokine receptors CXCR1, considering that it is activated by ROS production [87] and that the increase of inflammatory markers can modulate its transcription [88], and we found a significant high level of CXCR1 in cochlear lysates of styrene-treated animals with respect to controls (Fig. 4G, *n* = 3 animals/group; Student’s *t* test, *p* = 0.0002).

**Fig. 5:**
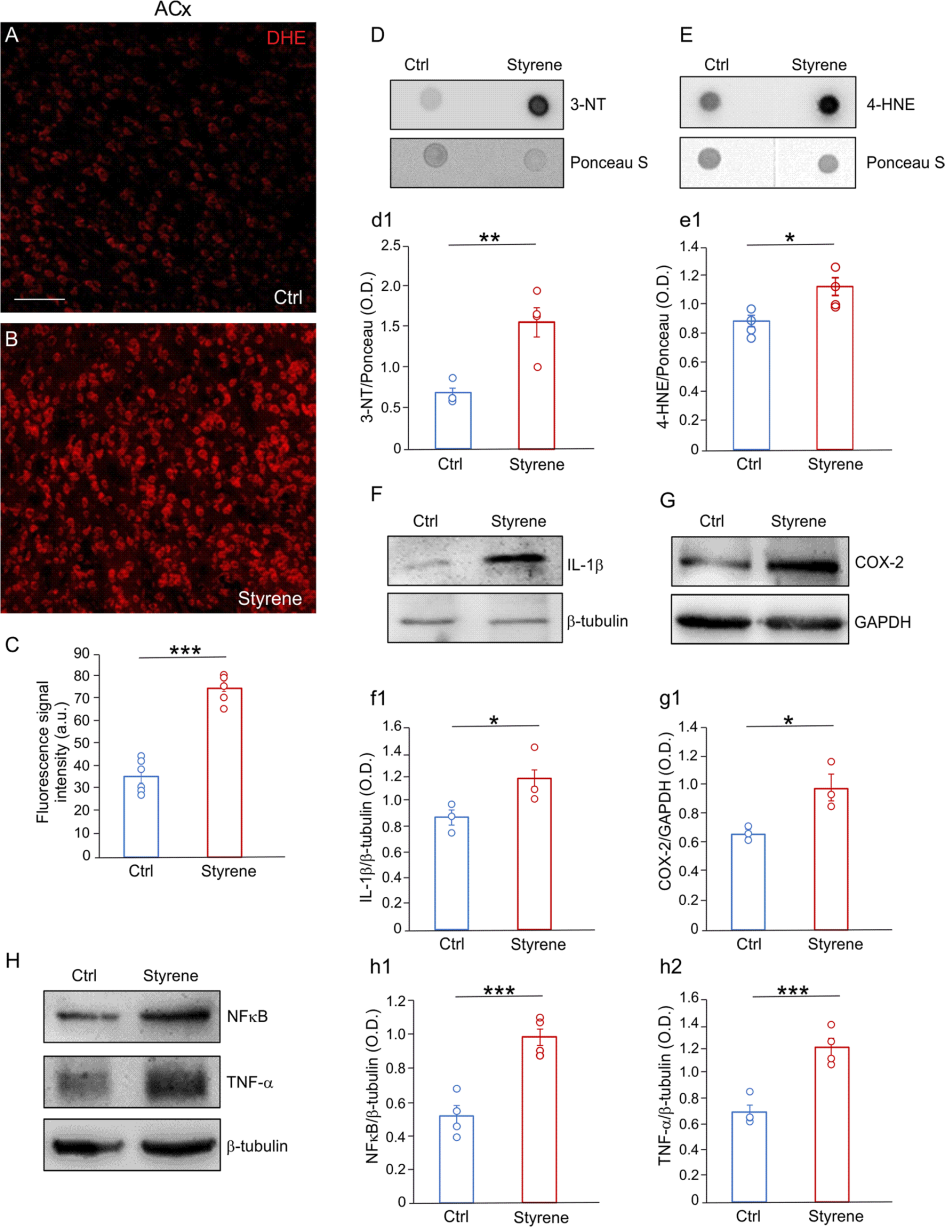
**Neurotoxic damage involves oxidative stress and neuroinflammation in the auditory cortex**. **A**, **B** DHE staining in brain coronal sections showing the auditory cortex (ACx) of control (**A**, Ctrl) and styrene-treated animals (**B**). **C** Histograms showing fluorescence intensity signal quantification. Data are expressed as mean ± SEM and are representative of three independent experiments from three animals/group. Scale bar: 100 μm. **D**, **E** Dot blots indicating an increase of protein tyrosine nitration (3-NT) and lipid peroxidation (4-HNE) in styrene-treated animals with respect to controls. **d1, e1** Histograms (means ± SEM) show semi-quantitative analyses of optical density (3-NT, *n* = 4 animals for each group; Student’s *t* test, *p* = 0.003; 4-HNE, *n* = 4 animals for each group; Student’s *t* test, *p* = 0.016). Equal protein loading was assessed by Ponceau S staining. **F**–**H** Representative western blots showing high levels of inflammatory markers, such as IL-1β (**F**), COX-2 (**G**), NFκB and TNF-α (**H**). **f1–h2** Graphs show the results of densitometric analyses on all samples for IL-1β (**f1**, *n* = 3 animals for each group; Student’s *t* test, *p* = 0.024), COX-2 (**g1**, *n* = 3 animals for each group; Student’s *t* test, *p* = 0.03), NFκB (**h1**, *n* = 4 animals for each group; Student’s *t* test, *p* = 0.0005) and TNF-α (**h2**, *n* = 4 animals for each group; Student’s *t* test, *p* = 0.001) normalized to the GAPDH or β-tubulin levels. Asterisks refers to significant differences between groups (*p < 0.05; **p < 0.01; ***p < 0.001)

Interestingly, in the ACx we also observed enhanced ROS amount in styrene-treated animals compared to controls, as shown by DHE assay on coronal brain sections (Fig. 5A, B) and confirmed by semi-quantitative analysis of fluorescence signal (Fig. 5C, *n* = 3 animals/group; Student’s *t* test, *p* < 0.0001). This was associated with high levels of 3-NT and 4-HNE, as shown by dot blot (Fig. 5D, E, 3-NT *n* = 4 animals/group; Student’s *t* test, *p* = 0.003; 4-HNE *n* = 4 animals/group; Student’s *t* test, *p* = 0.016). Western blot analyses of ACx lysates also revealed a significant high level of inflammatory markers, including IL-1β, TNF-α and NFκB, in styrene-treated animals with respect to controls (Fig. 5F, H, IL-1β, *n* = 3 animals/group; Student’s *t* test, *p* = 0.024; TNF-α *n* = 4 animals/group; Student’s *t* test, *p* = 0.001; NFκB *n* = 4 animals/group; Student’s *t* test, *p* = 0.0005). According to what observed in the cochlea, we found a significant high COX-2 protein level also in the ACx of Styrene group compared to Ctrl (Fig. 5G, *n* = 3 animals/group; Student’s *t* test, *p* = 0.03).

Collectively, these data demonstrate that the combined detrimental effect of oxidative stress and inflammation impinges on the auditory system of styrene-exposed rats, affecting both peripheral (cochlea) and central (ACx) regions.

### Macrophages activation in the cochlea

**Fig. 6:**
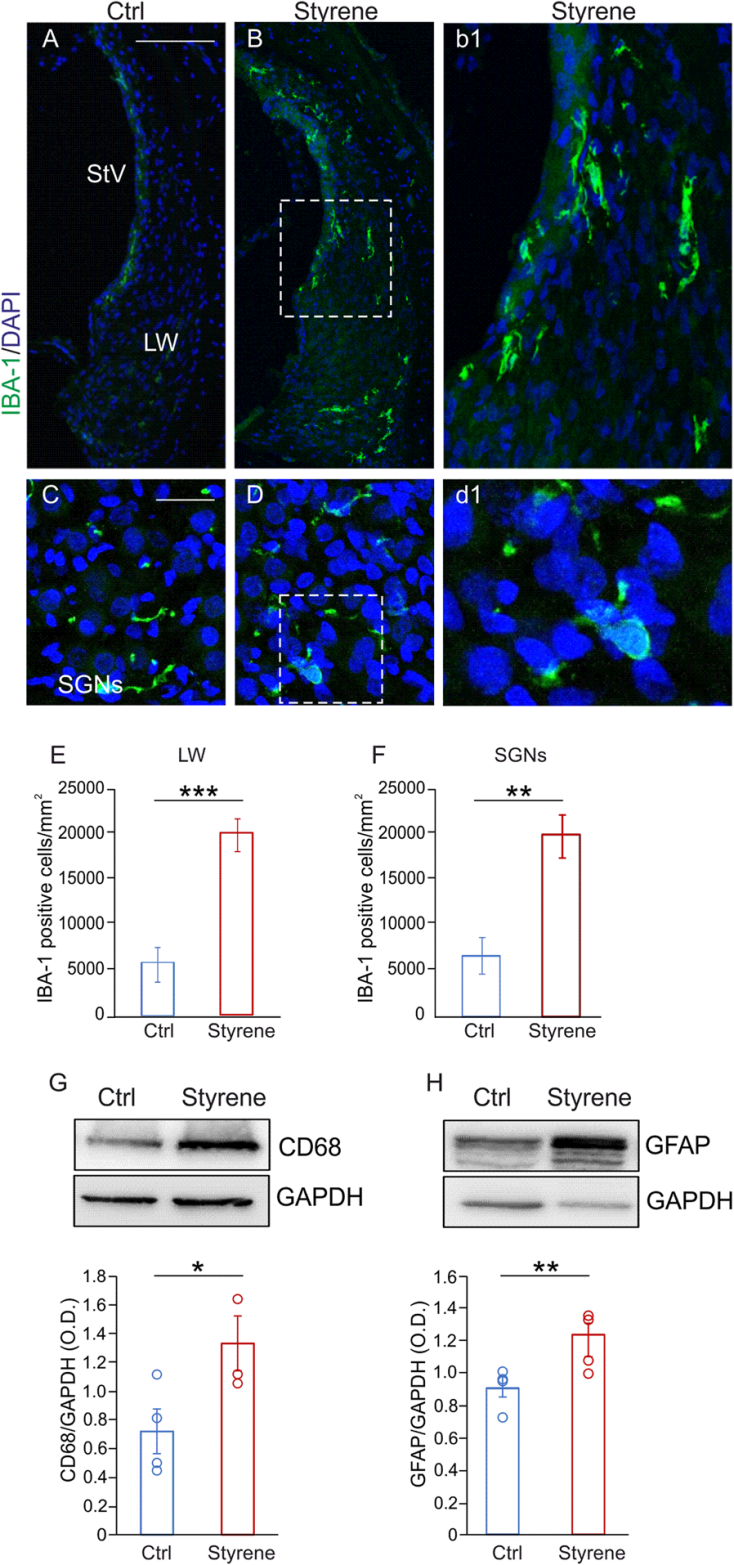
**Macrophages play a role in ototoxic damage induced by styrene. A**–**D** Images of cochlear sections showing high magnifications of the lateral wall (LW) with stria vascularis (SV; **A**, **B**) and spiral ganglion neurons (SGNs; **C**, **D**) stained with IBA-1, (green fluorescence) and DAPI (blue fluorescence). High magnifications show a strong IBA-1 fluorescence in the LW (**b1**) and SGNs (**d1**) in styrene cochlear samples. Scale bar: **A**, **B** 100 μm; **C**, **D** 50 μm. **E**, **F** Histograms showing IBA1-positive cell density in the LW (**E**) and in SGNs (**F**). Data are expressed as mean ± SEM and are representative of three independent experiments from three animals/group. **G**, **H** Representative western blots showing high levels of CD68 and GFAP in cochlear lysates in Styrene compared to Ctrl groups. Histograms (means ± SEM) show the optical density values (CD68 *n* = 3 cochleae for each group, *p* = 0.03; Student’s *t* test; GFAP *n* = 4 cochlea for each group; Student’s *t* test, *p* = 0.009) normalized to the corresponding total protein amount (GAPDH). Asterisks show statistical significance (*p < 0.05; **p < 0.01; ***p < 0.001)

Our immunofluorescence analyses in cochlear cryosection showed an increase of IBA-1 positive cells in samples of styrene-treated animals compared to controls (Fig. 6A–F), specifically in the lateral wall with stria vascularis (Fig. 6B, b1, E; Student’s *t* test, *p* = 0.0001) and SGNs (Fig. 6D, d1, F; Student’s *t* test, *p* = 0.002). Moreover, we found increased levels of the phagocytic marker CD68 in cochlear lysates of styrene-treated animals with respect to controls (Fig. 6G, *n* = 4 cochlea/group; Student’s *t* test, *p* = 0.03). Considering that supporting cells in the cochlear sensory epithelium, including Schwann cells and satellite cells, express GFAP [89], we also evaluated its expression in cochlear lysates, and we found a significant elevation of GFAP levels in styrene-treated animals, with respect to controls (Fig. 6H, *n* = 4 cochleae/group; Student’s *t* test, *p* = 0.009). Collectively, this data documented macrophages activation in the cochlea after styrene insult.

### Microglia and astrocytes characterization in the ACx

**Fig. 7:**
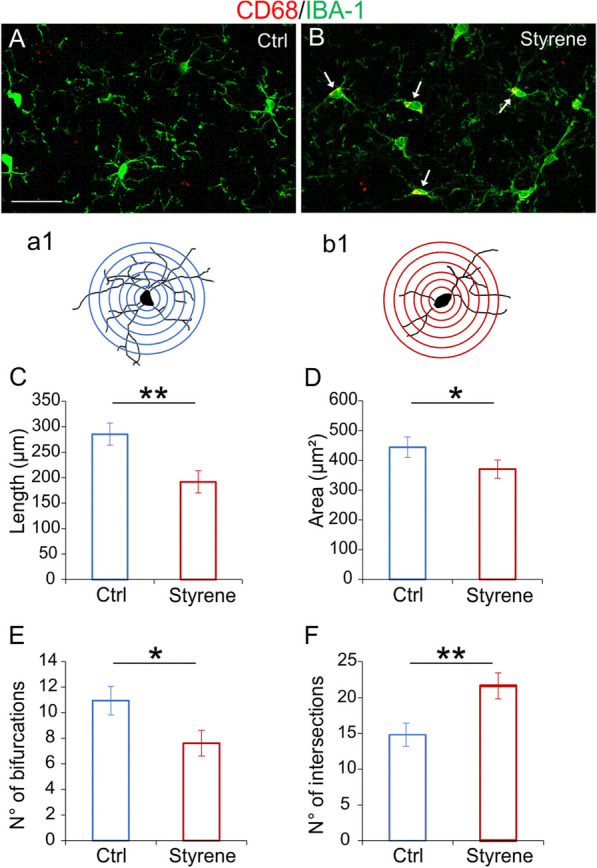
**Altered microglia morphology in the auditory cortex of styrene-treated animals. A**, **B** Representative images of brain coronal sections showing IBA-1 (green fluorescence) and CD68 (red fluorescence), expression in the auditory cortex (ACx) of control (**A**, Ctrl) and styrene-treated animals (**B**). Arrows in B indicate the co-localization of CD68 in IBA-positive cells. Scale bar: 50 μm. **a1, b1** Schematic representation of camera lucida drawings of single cell from control (**a1**) and styrene-treated animal (**b1**) analyzed with Sholl analysis. **C**–**F** Bar graphs (mean ± SEM) indicate differences in dendritic length (**C**, Student’s *t* test, *p* = 0.002), average of total surface area (**D**, Student’s *t* test, *p* = 0.017), total number of bifurcating nodes (**E**, Student’s *t* test, *p* = 0.02) and total number of dendritic intersections (**F**, Student’s *t* test, *p* = 0.007) of IBA1-positive cells in both experimental groups. Asterisks indicate significant comparisons (*p < 0.05; **p < 0.01)

**Fig. 8:**
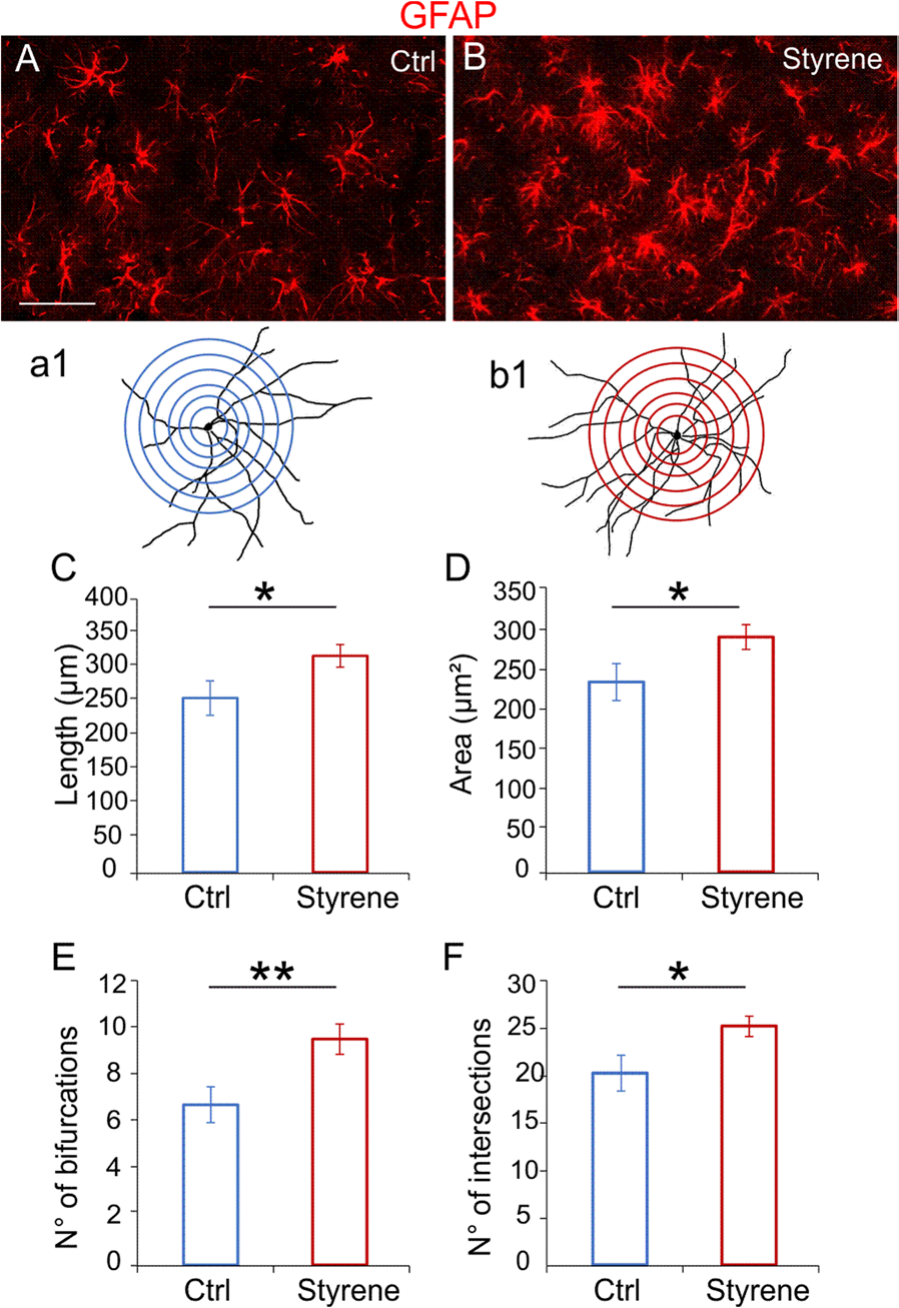
**Neurotoxic insult causes astrogliosis in the ACx. A**, **B** Representative images of brain coronal sections stained with GFAP (red fluorescence) showing the auditory cortex (ACx) of control (**A**, Ctrl) and styrene treated animals (**B**). Scale bar: 50 μm. **a1, b1** Schematic representation of a camera lucida drawing of a single GFAP-positive astrocyte from control (**a1**) and styrene-treated animal (**b1**) analyzed with Sholl analysis. **C**–**F** Bar graphs (mean ± SEM) indicate differences in total dendritic length (**C**, Student’s *t* test, *p* = 0.03), average of total surface area (**D**, Student’s *t* test, *p* = 0.03), number of bifurcating nodes (**E**, Student’s *t* test, *p* = 0.002) and number of total intersections (**F**, Student’s *t* test, *p* = 0.02) of astrocytes marked with GFAP in both experimental groups. Asterisks indicate significant differences between groups (**p < 0.01; ***p < 0.001)

In the ACx, we found an increase of CD68 expression in IBA-1 positive cells indicating macrophages/microglia activation following styrene toxic insult (Fig. 7A, B). Considering that functional alterations in microglia are usually associated with changes in their morphology [90], we further investigated the effects of the styrene administration on the structural features of glial cells. Specifically, microglia cells of styrene-treated animals showed shorter process length (Fig. 7C, Student’s *t* test, *p* = 0.002), diminished total area (Fig. 7D, Student’s *t* test, *p* = 0.017), reduced number of bifurcating nodes (Fig. 7E, Student’s *t* test, *p* = 0.02) and decreased number of intersections (Fig. 7F, Student’s *t* test, *p* = 0.007). Moreover, the increase of GFAP immunoreactivity in styrene-treated animals (Fig. 8A, B) was associated with morphological changes in astrocytes, with increased process length (Fig. 8C, Student’s *t* test, *p* = 0.03) and an increase of total area (Fig. 8D, Student’s *t* test, *p* = 0.03), number of bifurcating nodes (Fig. 8E, Student’s *t* test, *p* = 0.002) and number of intersections (Fig. 8F, Student’s *t* test, *p* = 0.03) in styrene-treated animals compared to controls.

Collectively, these data demonstrate that styrene toxic insult induced structural changes in glial cells, leading to a shift from “resting” to “active” morphology.

### Altered connexin and pannexin expression contributes to glial cell activation and to the oxidative/inflammatory damage

**Fig. 9:**
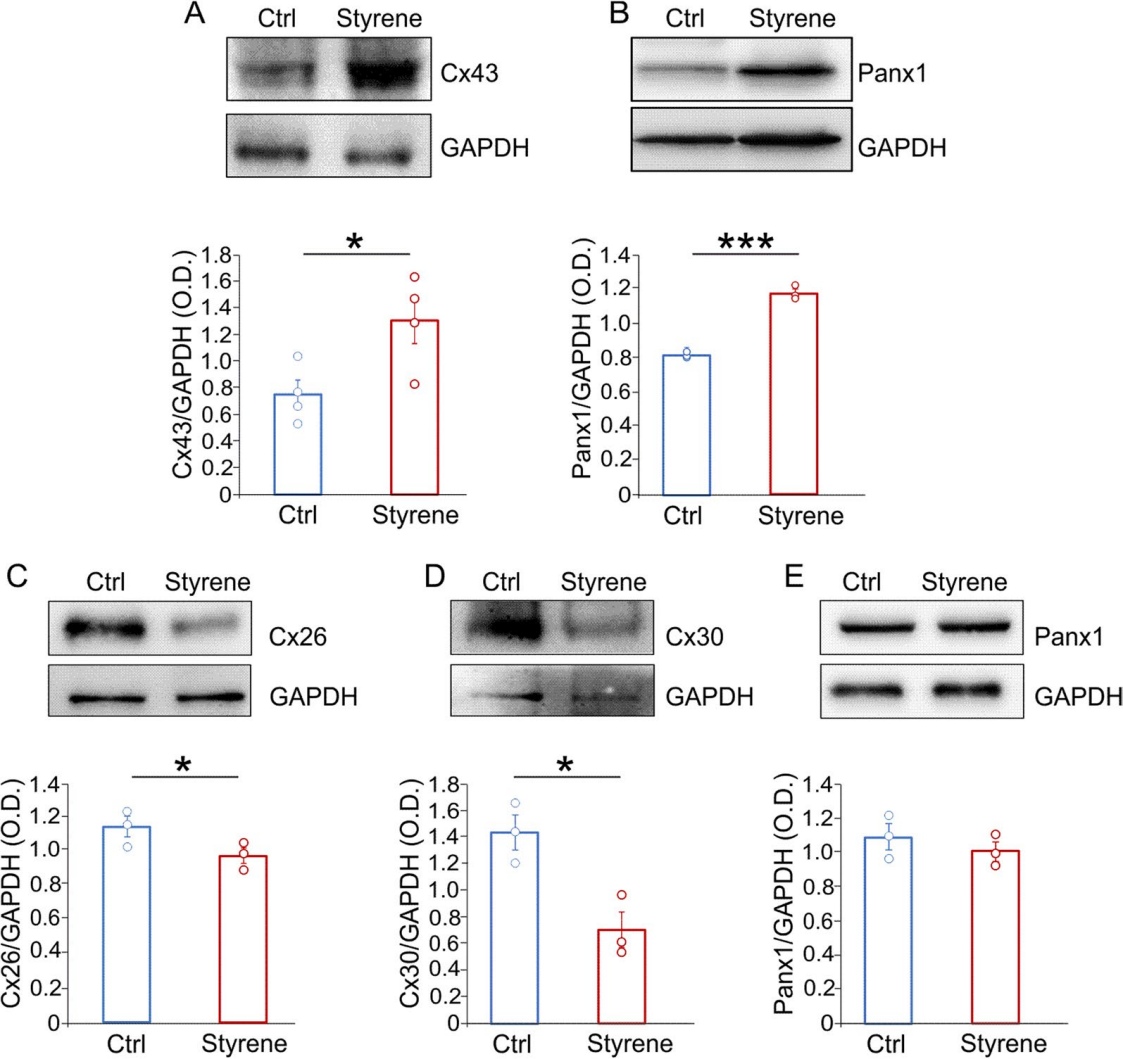
**Different alterations of connexin and pannexin expression in the cochlea and auditory cortex after the oto/neurotoxic insult.** **A**, **B** Representative bands of western blot showing high levels of Cx43 and Panx1 in the auditory cortex (ACx) of styrene-treated animals compared to controls. Bar graphs show optical density values in all samples (Cx43 *n* = 3 animals/group; Student’s *t* test, *p* = 0.03; Panx1 *n* = 3 animals/group; Student’s *t* test, *p* = 0.0001) normalized to the corresponding total protein levels (GAPDH). **C–E** Western blots showing expression levels of Cx26, Cx30 and Panx1 in cochlear lysates of styrene-treated animals compared to controls. Bar graphs show densitometric analyses (Cx26, *n* = 3 animals/group; Student’s *t* test, *p* = 0.04; Cx30, *n* = 3 animals/group; Student’s *t* test, *p* = 0.017; Panx1, *n* = 3 animals/group; Student’s *t* test, *p* > 0.05). Asterisks indicate significant differences between groups (*p < 0.05; ***p < 0.001)

Finally, we wondered if pannexons and gap junctions participate in the glial cell alterations reported above. In the ACx, we focused on Cx43, the major connexin isoform in the brain, and on Panx1. Our results showed a significant higher level of both Cx43 and Panx1 in brain samples of styrene-treated animals (Fig. 9A, B, Cx43 *n* = 3 animals/group; Student’s *t* test, *p* = 0.03; Panx1 *n* = 3 animals/group; Student’s *t* test, *p* = 0.0001). In the cochlea, we focused on Cx30 and Cx26, that are known to play a crucial role in cochlear physiology [91, 92]. Differently from what observed in the brain, we found a strong reduction of Cx30 and Cx26 in the cochlea of styrene-treated animals, compared with control specimens (Fig. 9C, D, Cx26: *n* = 3 samples/group; Student’s *t* test, *p* = 0.04; Cx30: *n* = 3 samples/group; Student’s *t* test, *p* = 0.01). On the other hand, the expression of Panx1 was similar comparing Ctrl vs Styrene group (Fig. 9E, *n* = 3 samples/group; Student’s *t* test, *p* > 0.05).

## Discussion

In this study, we explored the role of glial cells in the auditory system damage using a model of oto/neurotoxicity induced by styrene exposure. Indeed, even though several reports underlined the crucial role of microglia and astrocytes in mediating oxidative/inflammatory damage in brain disorders, including neurodegenerative diseases, the role of glial cells in the auditory system injury is still elusive. Here, we report that microglia and astrocytes mediate oxidative/inflammatory damage in the auditory system. Specifically, we demonstrated that the oto/neurotoxic insult induced by styrene increased oxidative stress and inflammation in both the cochlea and the ACx via macrophages and glial cell activation, involving altered expression of connexins and pannexin, probably dysregulating microglia/astrocyte networks.

First, we characterized our model of oto/neurotoxicity by assessing the detrimental effects of styrene exposure on both cochlea and ACx. In agreement with literature data [66, 67, 69], we found that styrene exposure induced a marked ototoxicity, as documented by the increase of auditory threshold associated with structural alterations in the cochlear sensory-neuronal compartment, such as decreased number of hair cell/neuronal afferent fiber contacts and decreased amplitude of ABR wave II, reflecting diminished number of neurons firing in the cochlear nuclei [70]. Interestingly, we also observed functional, morphological, and molecular alterations in the ACx of styrene-treated animals, with decreased basal synaptic transmission in the horizontal connections of auditory neurons, decreased number of dendritic spines and alterations in post-synaptic proteins, such as GluA1 phosphorylation. Specifically, phosphorylation at Ser845 GluA1 subunit is known to stabilize AMPA receptor at dendrites [93] and it is considered critical for sensory deprivation-induced homeostatic synaptic response and for experience-dependent synaptic plasticity [83, 84].

Several studies support the existence of a strong association between oxidative stress and inflammation in cochlear damage due to exogenous factors, including ototoxic injury. Indeed, when redox balance is altered, inflammatory response increases, triggering a vicious circle with a consequent rise of both ROS and pro-inflammatory cytokines [94–97]. The increase of oxidative stress observed not only in the cochlea but also in the ACx, confirms that the mechanism of action of styrene primarily involves mitochondrial damage and oxidative stress [98–101].

Looking for a cellular mechanism linking oxidative stress and inflammation, we focused on glial cells. Our analyses showed a high IBA-1 positive cells and GFAP level in the cochlea, paralleled to elevated of oxidative stress and inflammatory marker amount. Specifically, macrophages in the lateral wall and SGNs were activated, as revealed by the increased number of amoeboid cells with a pro-inflammatory and phagocytic phenotype. These findings agree with macrophages activation observed in different experimental models of cochlear damage, such as aminoglycoside ototoxicity, age-related hearing loss and noise exposure [38, 102–106]. Our western blot analyses also revelated high CD68 protein levels. Whether this was related to increased number of cells expressing CD68 in the cochlea, to inhibition of CD68 degradation, or to an increase in CD68 protein synthesis at the single-cell level, needs further investigations.

Activated glia cells can release several neurotoxic markers [107–109], including NO. The synthesis of NO is catalyzed by NO synthases (NOS) [110], such as iNOS. The latter is expressed in macrophages, microglia, astrocytes, and other cell types in response to inflammatory stimuli, LPS and cytokines [110, 111], and it has been found to be expressed by activated microglia [108, 111, 112]. Interestingly, in conjunction with higher protein tyrosine nitration and lipid peroxidation, as markers of oxidative stress, we found a significant high level of iNOS in cochlear lysates of styrene-treated animals. These data are also consistent with the elevated COX-2 level in the same samples, considering that COX-2 inhibitors can reduce iNOS expression in in vitro models of activated microglia [113]. The high level of the main inflammatory markers and of the chemokine receptor CXCR1, can be linked to the activation of glial cells. Indeed, it is known that microglia secrete inflammatory cytokines, such as tumor necrosis factor alpha (TNF-α), interleukin-1 beta (IL-1β), IL-6, and chemokines [114–116]. This can lead to the activation of microglia NADPH oxidase and to neuronal death, through the formation of peroxynitrite and iNOS production [117]. Moreover, the expression of pro-inflammatory cytokine TNF-α, can increase iNOS [118–120], and the chemokine receptor CXCR1 [121] in neuronal cells.

The involvement of microglia and astrocytes was also confirmed by immunofluorescence and morphological analyses on brain sections. Traditionally, the morphology of microglia has been classified in a “resting” state, characterized by highly branched morphology, and an “active” state, in which cells show an amoeboid form with decreased branch complexity [122, 123]. Our morphological analyses in ACx confirmed the presence of morphological changes documenting microglial activation after styrene exposure, including diminished branch length, diminished number of bifurcating nodes, intersections, and surface area. Moreover, the increased expression of CD68 indicates phagocytic activity [124]. Similar to microglia, reactive astrocytes exhibit several molecular and morphological features, including increased GFAP expression [72, 125]. Following an injury, reactive astrocytes increased in number and show altered morphology with hypertrophy of cellular processes [126, 127]. Accordingly, our data reports an increase of astrocytic branching complexity in the ACx of Styrene group, compared to controls.

Finally, considering that pro-inflammatory signaling occurs via gap junctions, hemichannels, and pannexons, favoring the shift between resting and active microglia near the injury site, we looked for connexin and pannexin expression in the cochlea and in ACx after styrene exposure. Interestingly, in the ACx we found an elevated level of Cx43 and Panx1, two proteins playing a crucial role in microglia and astrocyte coupling [51]. Indeed, when microglia become activated, the expression level of connexins, particularly Cx43, increases [49, 128, 129]. In physiological condition, astrocytes are strongly paired via gap junctions, expressing high levels of Cx43 [130]. However, decreased Cx43 amount have been observed in astrocytes cultured with resting microglia [56], whereas increased Cx43 expression was observed following LPS treatment [59]. Moreover, Cx43 and Panx1 functioning is also modulated by several inflammatory/toxic agents, including TNF-α, interferon gamma (IFN-γ) or amyloid-beta protein [129]. Similarly, Cx43 participates to ROS spreading among cells [131]. In addition, increased surface levels of Panx1 and Cx43 correlates with channel opening and microglial activation [132]. Finally, Panx1 channels activity can modulate microglial morphology and microglia/neurons interactions [133].

In the cochlea we studied Panx1 and, among connexins, we focused on the two main connexins playing a crucial role in inner ear physiology, i.e., Cx26 and Cx30 [91, 134]. In contrast to what observed in the brain, we found a downregulation of connexins and no changes in pannexin expression in the cochlea of styrene-treated animals compared to controls. This different pattern of connexin/pannexin modulation in the cochlea and ACx seems to be inconsistent; however, it should be considered the different functional expression of connexins and pannexins in the brain and in the inner ear. Indeed, while Cx43 and Panx1 have been related to microglia activation in the CNS [51], Cx30 and Cx26 are essential for hearing function, given that deletion of the *GJB2* and *GJB6* genes, encoding for Cx26 and Cx30, respectively, is the main cause of non-syndromic sensorineural hearing loss in the Mediterranean population [135–137]. Moreover, in animal models of age-related hearing loss it has been demonstrated that oxidative stress can downregulate cochlear Cx26 and Cx30 expression [138, 139]. On the other hand, decreased Cx26 and Cx30 levels can exacerbate redox imbalance, impairing the endogenous antioxidant enzyme efflux in the cochlear sensory epithelium [92, 140]. Thus, our data seem to be consistent with the increased oxidative stress observed in styrene-treated samples. Notably, we did not find a significant difference in Panx1 cochlear level between styrene-exposed and control animals. However, the role of Panx1 in the cochlea is controversial, with some studies suggesting that altered Panx1 expression can cause hearing loss [141, 142] and others supporting the idea that Panx1 is dispensable for hearing [143, 144].

## Conclusions

Collectively, our data indicate that the oto/neurotoxic effects of styrene rely on oxidative/inflammatory damage occurring in both the cochlea and ACx. In both auditory structures activated macrophages, microglia and astrocytes mediate the crosstalk between oxidative stress and inflammation, also via the altered expression of connexins and pannexins. Specifically, in the cochlea the ototoxic effect of styrene induces an increase of oxidative stress with diminished connexin expression (probably exacerbating redox imbalance), macrophages activation and increased inflammation. In the ACx, the neurotoxic insult causes oxidative stress, microglia, and astrocyte activation with increased neuroinflammation and decreased connexin and pannexin expression. This probably causes alterations in microglia/astrocytes coupling, exacerbating neuroinflammation.

### Supplementary Information

**Additional file 1.** **Supplementary Table 1:** List of abbreviations.

**Additional file 2.** Arrive guidelines.

## Data Availability

The data included in the article are available from the corresponding author.
