# Supplementary material for: Oxidative stress and inflammation cause auditory system damage via glial cell activation and dysregulated expression of gap junction proteins in an experimental model of styrene-induced oto/neurotoxicity
Source: J Neuroinflammation. 2024 Jan 4;21:4. doi: 10.1186/s12974-023-02996-3 (PMC10765700; doi:10.1186/s12974-023-02996-3)
Supplement: Supplementary file 1 — Additional file 1. Supplementary Table 1: List of abbreviations. [file 12974_2023_2996_MOESM1_ESM.docx]

| **ACx** | Auditory Cortex |
| --- | --- |
| **aCSF** | Artificial cerebrospinal fluid |
| **3-NT** | 3-nitrotyrosine |
| **4-HNE** | 4-hydroxynonenal |
| **ABR** | Auditory brainstem responses |
| **A–I** | Amplitude-intensity curve |
| **CNS** | Central Nervous System |
| **COX-2** | cyclooxygenase-2 |
| **CtBP2** | C-terminal-binding protein 2 |
| **Cx26** | Connexin 26 |
| **Cx30** | Connexin 30 |
| **Cx43** | Connexin 43 |
| **CXCR1** | Chemokine Receptor 1 |
| **DAPI** | 4′,6-Diamidino-2-Phenylindole |
| **DHE** | Dihydroethidium |
| **fEPSPs** | Field excitatory post-synaptic potentials |
| **GFAP** | Glial fibrillary acidic protein |
| **GJB2/6** | Gap junction protein beta 2/6 |
| **GJCs** | Gap junctions |
| **HCs** | hemichannels |
| **IBA-1** | Ionized calcium binding adaptor molecule 1 |
| **IHCs** | Inner hair cells |
| **IL-1β** | Interleukin-1 beta |
| **iNOS** | Inducible nitric oxide synthase |
| **LW** | Lateral wall |
| **NF200** | Neurofilament 200 |
| **NF-κB** | Nuclear factor-κB |
| **oC** | Organ of Corti |
| **OHCs** | Outer hair cells |
| **Panx1** | Pannexin 1 |
| **ROS** | Reactive oxygen species |
| **RT** | Room temperature |
| **SGNs** | Spiral ganglion neurons |
| **SPL** | Sound pressure level |
| **SV** | Stria vascularis |
| **TNF-α** | Tumor Necrosis Factor-α |

**Supplementary Table 1: list of abbreviations.**
