# Supplementary material for: Oxidative stress and inflammation cause auditory system damage via glial cell activation and dysregulated expression of gap junction proteins in an experimental model of styrene-induced oto/neurotoxicity
Source: J Neuroinflammation. 2024 Jan 4;21:4. doi: 10.1186/s12974-023-02996-3 (PMC10765700; doi:10.1186/s12974-023-02996-3)
Supplement: Supplementary file 2 — Additional file 2. Arrive guidelines. [file 12974_2023_2996_MOESM2_ESM.pdf]

ARRIVE GUIDELINES	
**Study design**	For each experiment, brief details of study design have been provided in Result section and in Figure legends, including:<br>1. the groups being compared;<br>2. the experimental unit.
**Sample size**	a. The number of experimental units and the total number of animals used have been provided in Material and methods, Results and Figure legends.<br>b. Explanation of how the sample size was decided has been reported in Statistical analysis section.
**Inclusion and exclusion criteria**	a. The exclusion/inclusion criteria set, was baseline normal hearing and normal Preyer's reflex at the beginning of the study (as reported in Material and Method section).<br>b. No animals have been excluded.<br>c. For each analysis, the exact value of n in each experimental group has been provided (see Results and Figure legends).
**Randomisation**	a. We used online random number generator (https://www.graphpad.com/quickcalcs/randomize1/) to allocate experimental units to control and treatment groups.<br>b. the strategy used to minimize potential confounders (animal housing, sex, age, tissue processing) have been considered.
**Blinding**	Experiments were conducted in blind. Experimenters always have access to the group or treatment codes, but they do not know the correspondence between group and treatment before the end of the study.
**Outcome measures**	Outcome measures have been reported in Material and Method and Result sections.
**Statistical methods**	All statistical method used have been reported in Statistical analysis section.
**Experimental animals**	Relevant information about animals (including species, strain, sex, age, housing) have been provided in Materials and Methods.
**Experimental procedures**	For each experimental group, including controls, procedures have been described in Materials and Methods, including:<br>- what was done, how it was done and what was used;<br>- when and how often<br>- why (a rationale for each procedure has been reported).
**Results**	In Result and in Figure legends, we reported for each experiment a summary/descriptive statistic (including F values and p level).

**Ethical statement**	Information about the ethical review committee and ethical approval have been provided in Material and Methods.
**Housing and husbandry**	This information has been included in Materials and Methods.
**Animal care and monitoring**	According to the Italian Ministery of Health and to the Catholic University Ethical committee, animal monitoring has been performed by the Animal Facility staff of our Institution, following guidelines reported in Clinical Scores to promote continuity of good care" (PMID: 25957286; PMID: 22822473).<br>The humane endpoints established for the study were set following "How to determine humane endpoints for research animals." (Lab Anim (NY). 2016 J;45(1):19. doi: 10.1038/laban.908; https://www.humane-endpoints.info/en).<br>All information about animal care and monitoring and humane endpoint have been reported in the Prot. 1F295.116, approved by the Italian Department of Health (*Ministero della Salute*).
**Data access**	A data availability statement has been provided.
**Declaration of interests**	a. No potential conflict of interests has been declared.<br>b. List of all funding sources has been provided.
